# Evaluation of the Effect of Coronal Flaring on SWEEPS Laser-Activated Irrigation in Root Canals with Different Curvatures: A Confocal Laser Scanning Microscopy Study

**DOI:** 10.3390/medicina62091737

**Published:** 2026-09-09

**Authors:** İbrahim Sevinç, Esma Dinger

**Affiliations:** Department of Endodontics, Faculty of Dentistry, Bolu Abant Izzet Baysal University, Bolu 14030, Turkey

**Keywords:** confocal laser scanning microscopy, coronal flaring, root canal curvature, shock wave-enhanced emission photoacoustic streaming (SWEEPS)

## Abstract

*Background and Objectives:* The aim of this study was to evaluate the penetration of laser-activated irrigation solutions into dentinal tubules in root canals with different degrees of curvature and different coronal flaring. *Materials and Methods:* Seventy-six maxillary first molar teeth previously extracted for various reasons were included in the present study. The specimens were divided into two main groups according to distal root canal curvature, determined using the Schneider method: straight (<10°) and curved (20–40°). These groups were further classified into two subgroups according to whether a coronal flaring procedure was performed (*n* = 19). Following root canal preparation of all specimens, final irrigation was performed using a laser activation method and an irrigation solution prepared with the fluorescent dye Rhodamine B. Horizontal apical, middle, and coronal sections of 1 ± 0.1 mm thickness were obtained from the specimens at 2 mm, 5 mm, and 8 mm from the apical foramen, respectively. The effect of the irrigation solution on dentinal tubule penetration in the root canals was evaluated in the obtained sections using confocal laser scanning microscopy. Following evaluation, the mean and maximum penetration distances into the dentinal tubules were measured. The obtained data were statistically analyzed using Generalized Estimating Equations and Bonferroni correction for multiple comparisons, with the significance level set at *p* < 0.05. *Results:* The analysis revealed that only the region factor had a statistically significant effect on mean penetration values (*p* < 0.001), whereas canal curvature (*p* = 0.010), coronal flaring (*p* = 0.002), and region (*p* < 0.001) had statistically significant effects on maximum penetration values. However, none of the two-way or three-way interactions were significant (*p* > 0.05). *Conclusions:* Coronal flaring did not affect mean dentinal tubule penetration but increased maximum penetration depth. Maximum penetration was higher in straight canals than in curved canals, while both mean and maximum penetration were significantly affected by the root canal region.

## 1. Introduction

The primary aim of endodontic treatment is to eliminate the etiology of the lesion through effective chemomechanical cleaning and shaping of the root canal system. However, the morphological structure of root canals is not always suitable for simple and effective preparation. Various anatomical variations complicate root canal treatment [1]. Root canal morphology includes significant anatomical challenges, including lateral canals, accessory canals, isthmuses, apical deltas, and dentinal tubules.

In maxillary molars, anatomical variations may include differences in root and root canal morphology, including the presence of additional canals, and their prevalence may vary among different populations and geographic regions. The frequently occurring second mesiobuccal canal (MB2), isthmuses, and variations in canal configuration within the mesiobuccal root of maxillary first molars may constitute additional anatomical variables [2]. In the present study, the distobuccal canals of maxillary first molars, which predominantly exhibit Vertucci Type I root canal morphology and show less anatomical variation than the mesiobuccal root, were selected to ensure experimental standardization and minimize the influence of anatomical variations on the evaluated parameters [3].

In addition to these anatomical challenges, root canals with severe curvature also present a challenging condition for the shaping and disinfection of the root canal system [4]. The first step in the treatment of teeth with curved root canals is the radiographic detection of existing root canal curvatures and careful examination of preoperative radiographs. Many attempts have been made to measure the degree of curvature on radiographs. The most commonly preferred of these is the Schneider method [5,6,7]. According to the Schneider method, teeth with a root canal curvature of 5° or less are categorized as straight, those with a curvature of 10–20° as moderate, and those with a curvature of 25–70° as severe [8]. These categorical thresholds describe the conventional Schneider classification; however, predefined curvature ranges that do not strictly correspond to these categories have been used in experimental studies to standardize and clearly distinguish study groups [9,10].

Coronal flaring is an important preparation step that provides easier access to the apical region by enlarging the cervical and middle thirds of the root canal system before apical preparation, facilitates the penetration of irrigation solutions, removes debris more effectively, and reduces stress on the canal instrument during instrumentation [11,12]. A study concluded that performing coronal flaring initially reduced root canal curvature [13].

Although root canal preparation is one of the fundamental stages of root canal treatment, mechanical preparation alone is known to be insufficient to ensure effective cleaning and disinfection of the root canal system. Therefore, chemical irrigation and activation of irrigation solutions are critically important for achieving effective disinfection of the root canal system [14]. Considering the complex root canal anatomy and the limited capacity of irrigation solutions to disinfect the root canal system three-dimensionally, laser-activated irrigation techniques are considered an important approach for increasing the effectiveness of endodontic treatment [15]. Photon-induced photoacoustic streaming (PIPS) is a laser activation technique that activates irrigation solutions commonly used in endodontics with a low-energy laser (erbium:YAG laser-Er:YAG). PIPS triggers a single laser pulse with a square waveform during each emission cycle [16]. Shock wave-enhanced emission photoacoustic streaming (SWEEPS) is a technique in which pulsed laser light is emitted into the solution in the access cavity through the fiber tip of a laser handpiece positioned in the access cavity containing irrigation solutions. SWEEPS uses synchronized pairs of ultrashort pulses at an optimal time interval to accelerate the collapse of laser-induced bubbles. This pulse characteristic results in increased solution flow and shock wave emission even in the narrowest parts of the root canal [17,18,19].

Previous studies have investigated the effects of coronal flaring, root canal curvature, and activated irrigation on various aspects of root canal preparation and irrigant penetration. However, to the best of our knowledge, no study has specifically evaluated the combined effects of root canal curvature and coronal flaring on dentinal tubule penetration during SWEEPS activation. Although coronal flaring is routinely recommended to facilitate instrument use, its effect on the hydrodynamic efficiency of SWEEPS activation in curved canals remains unknown. Understanding this relationship is thought to enable clinicians to maximize irrigant delivery without increasing apical flaring. The aim of this study was to evaluate the effects of root canal curvature, coronal flaring, and root canal region, as well as their interactions, on the mean and maximum dentinal tubule penetration of a laser-activated irrigation solution during SWEEPS activation. The null hypothesis (H0) was that root canal curvature, coronal flaring, and root canal region, as well as their interactions, would have no significant effect on the mean or maximum dentinal tubule penetration of the laser-activated irrigation solution.

## 2. Materials and Methods

### 2.1. Ethical Approval and Sample Size Calculation

This study was approved by the Bolu Abant Izzet Baysal University Non-Interventional Clinical Research Ethics Committee with decision no. 2025/306 dated 22 July 2025.

The sample size was calculated using G*Power 3.1 based on an F test for repeated-measures ANOVA with between-subject factors, considering four experimental groups and three repeated regional measurements [20]. An effect size of f = 0.432 was calculated from the descriptive data reported in Table 3 of the reference study [21]. With an α level of 0.05, a statistical power of 95%, and an assumed correlation of 0.50 among repeated measurements, the minimum required sample size was calculated as 68 specimens (17 per group). After accounting for a potential 10% specimen loss during the experimental procedures, the target sample size was increased to 76 specimens, corresponding to 19 specimens per experimental group.

### 2.2. Selection of Specimens and Formation of Study Groups

The study included 76 three-rooted maxillary first molars with a single distal root exhibiting Vertucci Type I canal morphology. The teeth had previously been extracted for orthodontic, traumatic, or periodontal reasons from adult patients aged 18–60 years who presented to the Department of Oral and Maxillofacial Surgery, Faculty of Dentistry, Bolu Abant Izzet Baysal University.

Periapical radiographs were obtained to determine the root canal curvature and canal anatomy of the maxillary first molar teeth to be used in this study. Periapical radiographs were taken with the film positioned parallel to the long axis of the teeth, with the film and tooth surfaces positioned as close to each other as possible, and with the X-rays directed perpendicular to the teeth. All images were acquired under standardized conditions, maintaining a fixed distance of 27 cm between the film and the X-ray source and using the same exposure time [22]. All radiographs were obtained by a single experienced clinician to ensure consistency and standardization of the radiographic procedures.

Root canal curvatures were calculated from the periapical radiographs according to the Schneider method using the “Angle Tool” feature of ImageJ (ImageJ 1.54g; National Institutes of Health, Bethesda, MD, USA) [23,24].

To ensure standardization among the maxillary first molar teeth included in this study, the crown-root length between the distal root apex and the highest point of the crown was standardized to 20 ± 2 mm, and the root length was standardized to 12 ± 1 mm [25,26,27].

**Figure 1:**
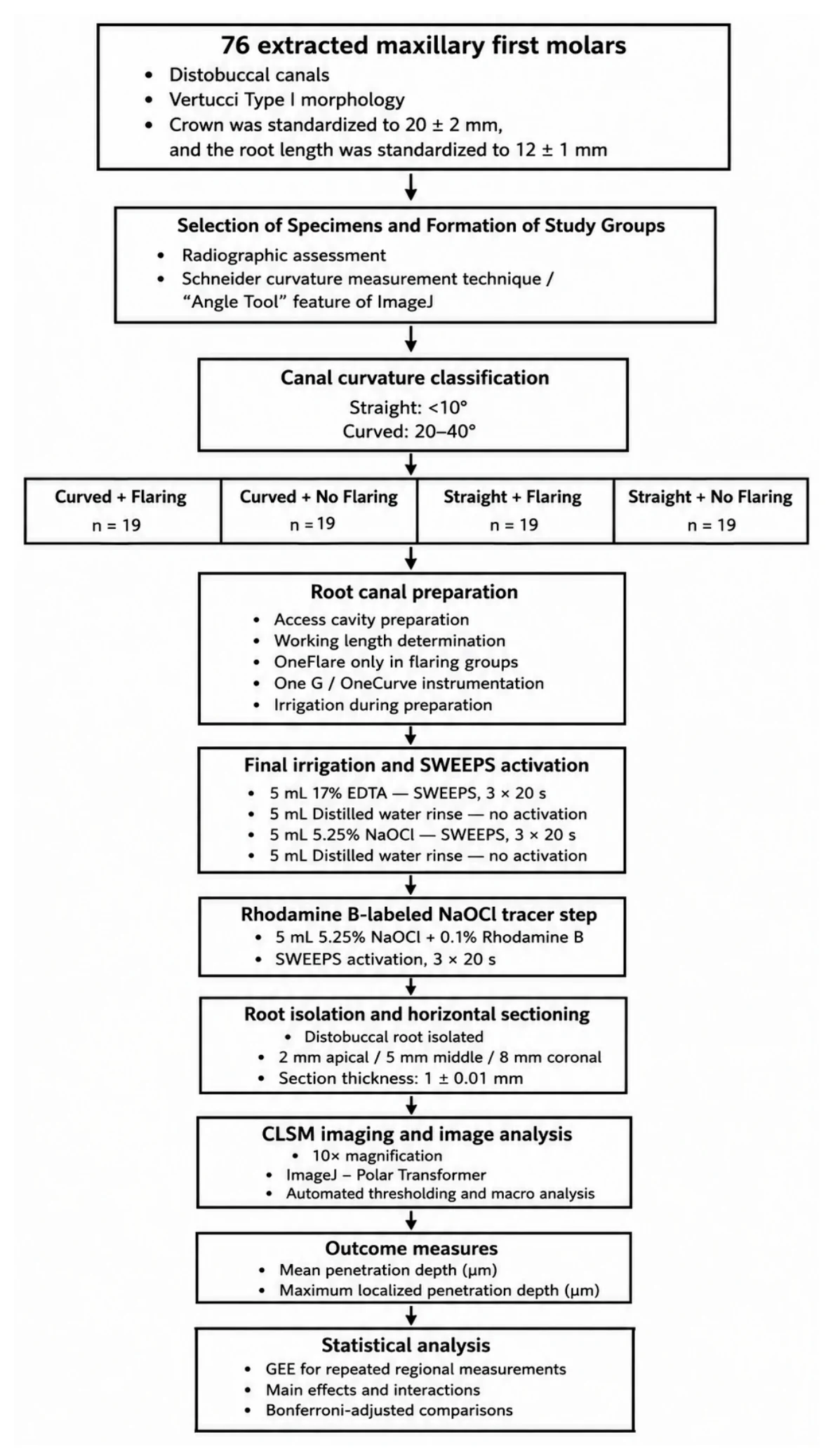
Flowchart of specimen selection, experimental grouping, root canal preparation, final irrigation protocols and activation, CLSM evaluation, and statistical analysis.

A total of 76 three-rooted maxillary first molar teeth with a single distal root and Vertucci Type I canal morphology in the distal root were divided into two groups according to the degree of curvature before the preparation procedure: teeth with curved root canals and teeth with straight root canals [27,28]. For experimental grouping, canals with curvature angles <10° were assigned to the straight canal group, whereas those with curvature angles between 20° and 40° were assigned to the curved canal group. These predefined ranges were selected to provide a clear separation between the experimental groups. The 20–40° range was selected based on previous experimental studies that used this interval for the evaluation of curved root canals [9,10]. Following curvature classification, the specimens within each curvature category were equally allocated to subgroups with and without coronal flaring using a computer-generated random number sequence (*n* = 19) (Figure 1). Canal curvature was assessed before instrumentation and was used for the initial classification of the specimens; post-preparation canal curvature was not reassessed.

A1: Teeth with Curved Root Canals (20–40°), coronal flaring (+).

A2: Teeth with Curved Root Canals (20–40°), coronal flaring (−).

B1: Teeth with Straight Root Canals (<10°), coronal flaring (+).

B2: Teeth with Straight Root Canals (<10°), coronal flaring (−).

### 2.3. Root Canal Preparation, Final Irrigation, and Activation Procedures

Access cavities were prepared using a cylindrical flat-end diamond bur (C 837-314-012-8; 1.2 mm diameter; G & Z Instrumente GmbH, Lustenau, Austria) at 300,000 rpm using a high-speed handpiece under air and water cooling. A #10 K-type file (EndoArt, Inci Dental, Istanbul, Türkiye, 2025) was inserted into the distal canal to confirm apical patency, and the working length was determined as 1 mm shorter than the point at which the file emerged from the apical foramen.

In the groups subjected to coronal flaring, root canal preparation was performed using OneFlare (Micromega, Besancon, France, 2024), OneG (Micromega, Besancon, France, 2024), and OneCurve 25/.04 (Micromega, Besancon, France, 2024) files with a Woodpecker Ai-Motor (Guilin Woodpecker Medical Instrument Co., Ltd., Guilin, China). Considering the crown-root lengths, preparation with the OneFlare coronal flaring file was performed by advancing 5–6 mm from the canal orifice to remove irregularities in the coronal and middle thirds [29,30]. The expected canal diameter at the coronal flaring level was determined according to the apical diameter and taper of the OneFlare instrument in relation to the distance that the instrument was advanced within the root canal. In this direction, coronal diameter was standardized in groups that underwent flaring preparation.

In the groups without coronal flaring, the root canals were prepared sequentially using OneG (Micromega, Besancon, France) and OneCurve 25/.04 (Micromega, Besancon, France) files with a Woodpecker Ai-Motor (Guilin Woodpecker Medical Instrument Co., Ltd., Guilin, China, 2022). During preparation, irrigation was performed with 5.25% NaOCl solution (Saver, Prime Dental, Maharashtra, India, 2025) using a 30G side-vented needle (Medic, Shanghai, China, 2024) to remove debris from the root canals.

In all specimens, laser irrigation activation was performed in SWEEPS mode using an Er:YAG laser device (LightWalker, Fotona, Ljubljana, Slovenia) during the activation procedures throughout the final irrigation. Activation was performed by placing a 14 mm long, 600 μm diameter radial fiber tip (SWEEPS 600/14; SWEEPS, Fotona, Ljubljana, Slovenia, 2023) in the pulp chamber, using the method applied by Er Karaoglu G. et al., in three 20 s cycles (a total of 1 min) at settings of 20 mJ, 15 Hz, and 0.30 W, without air or water spray [23,31,32,33].

During the final irrigation protocol, 5 mL of 17% EDTA (Microvem, Altun Sterilization and Medical, Konya, Türkiye), was first activated using the SWEEPS mode for three 20 s cycles (total activation time: 60 s). This was followed by irrigation with 5 mL of distilled water without activation. Subsequently, 5 mL of 5.25% NaOCl (Saver, Prime Dental, Maharashtra, India, 2025) was activated using the same SWEEPS protocol for three 20 s cycles (total activation time, 60 s), followed by a final rinse with 5 mL of distilled water without activation. The canals were subsequently dried with paper points.

### 2.4. Staining of Specimens with a Solution Prepared Using Rhodamine B

The Rhodamine B-containing solution was prepared by adding 0.1 g (0.1%) of Rhodamine B (Ege Nanotek Kimya Sanayi, İzmir, Türkiye, 2025), weighed using a precision balance, to 100 mL of 5.25% NaOCl and mixing homogeneously. The solution was freshly prepared to avoid potential chemical interactions [34,35]. A solution containing fluorescent Rhodamine B (Ege Nanotek Kimya Sanayi, İzmir, Türkiye), was subsequently applied to enable measurement of the maximum and mean penetration depths of the irrigation solution into the dentinal tubules. CLSM detection was based on Rhodamine B fluorescence and therefore represented the distribution of the fluorescently labeled NaOCl solution rather than direct detection of NaOCl itself.

During the application procedure, 5 mL of solution containing 0.1% Rhodamine B was activated to fluorescently stain the dentinal tubules using an Er:YAG laser device (LightWalker, Fotona, Ljubljana, Slovenia) in SWEEPS mode for three 20 s cycles (a total of 1 min) at settings of 20 mJ, 15 Hz, and 0.30 W. After negative aspiration from the canals, the canals were dried using paper points (EndoArt, Inci Dental, Istanbul, Türkiye, 2024) [32,36,37]. All root canal preparation and irrigation activation procedures were performed by the same experienced operator to minimize experimental variability.

### 2.5. Confocal Laser Scanning Microscopy Imaging

**Figure 2:**
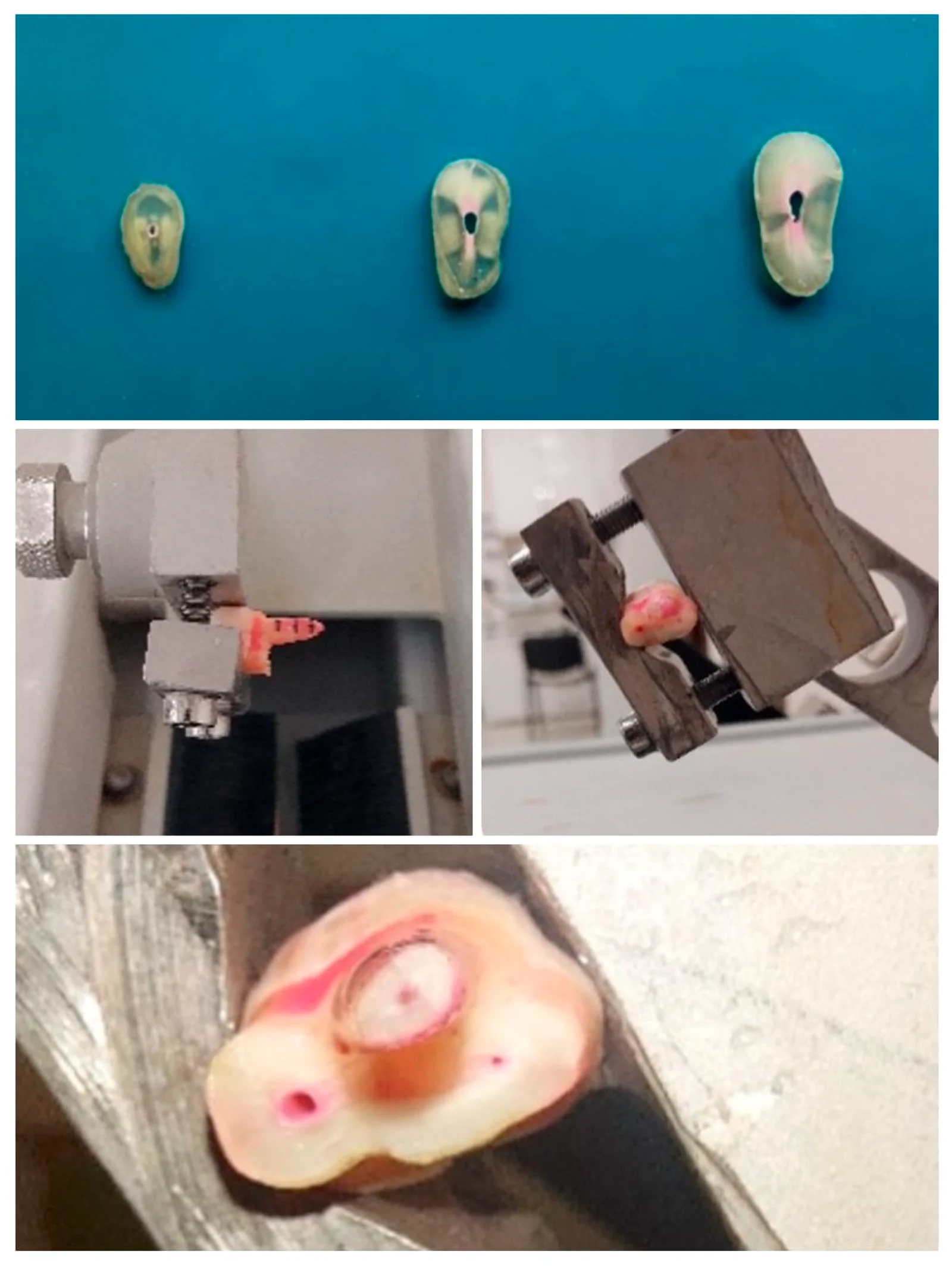
Procedural steps for obtaining horizontal sections from the distobuccal root.

Following completion of the irrigation activation procedures, the palatal and mesial roots were removed from the furcation level using a cylindrical flat-end diamond bur (C 837-314-012-8; 1.2 mm diameter; G & Z Instrumente GmbH, Lustenau, Austria, 2025) at 300,000 rpm using a high-speed handpiece under air and water cooling, according to the method applied in a previous study, to allow sections to be obtained from the specimens [38]. The distal roots were then isolated, and the 2 mm, 5 mm, and 8 mm levels from the apical foramen were measured and marked for sectioning. Horizontal sections with a thickness of 1 ± 0.1 mm were obtained using a precision cutting device (IsoMet 1000 Precision Saw, Buehler, Lake Bluff, IL, USA), with both surfaces of each section parallel to each other (Figure 2) [39].

**Figure 3:**
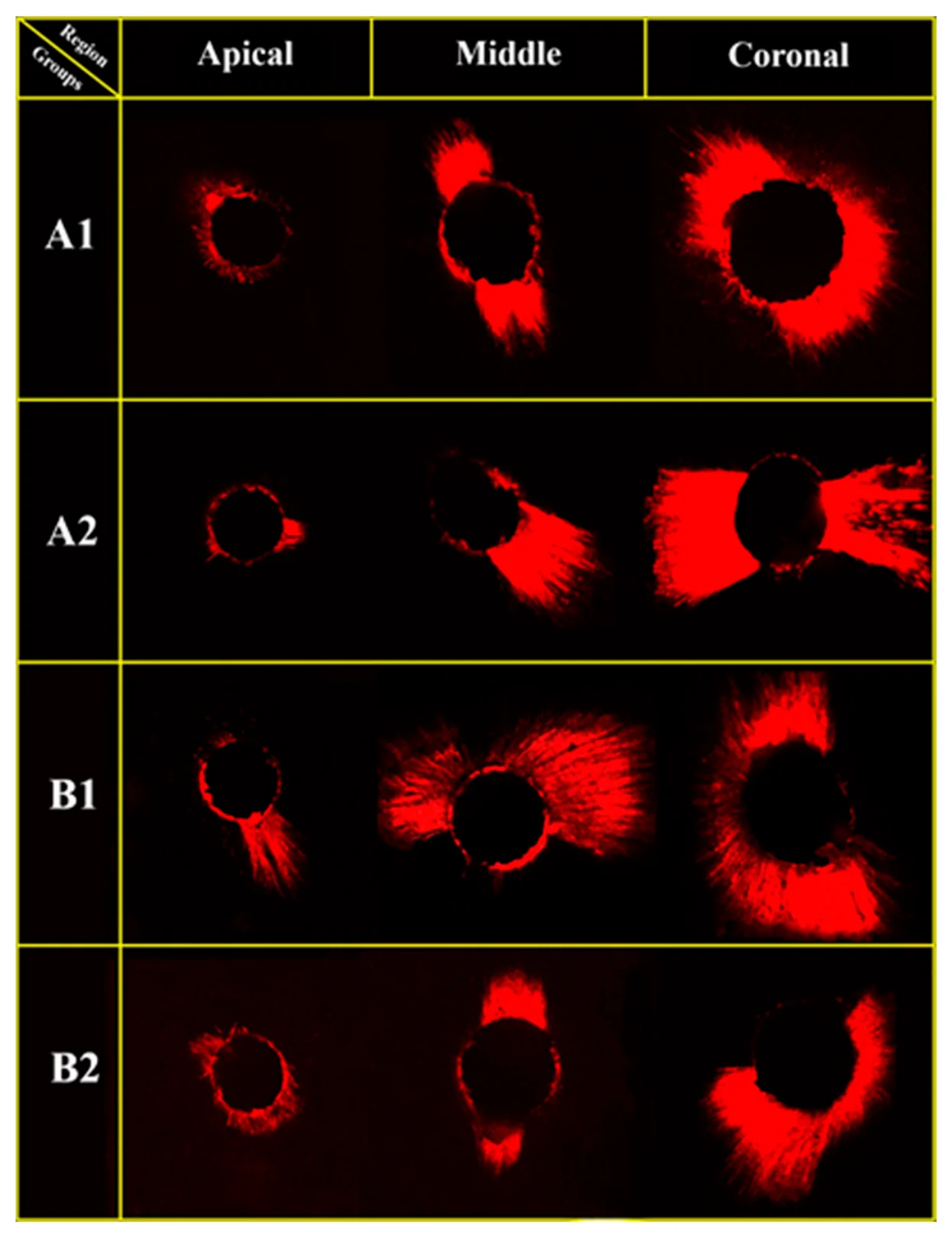
Images of the apical, middle, and coronal thirds obtained using a confocal laser scanning microscope.

The horizontal dentin sections obtained using the precision cutting device were examined under 10× magnification using a Nikon C2 Confocal Laser Scanning Microscope (CLSM) (Nikon Corporation, Tokyo, Japan). Images with a resolution of 512 × 512 pixels were obtained from the specimens. CLSM was preferred for measuring dentinal tubule penetration because of the reproducibility of the images (Figure 3), and the detailed information it provides at 10× magnification in specimens stained with Rhodamine B [40].

Mean penetration distance (µm) and maximum penetration distance (µm) into the dentinal tubules were measured from the acquired raw images using ImageJ software (ImageJ 1.54g; National Institutes of Health, Bethesda, MD, USA).

**Figure 4:**
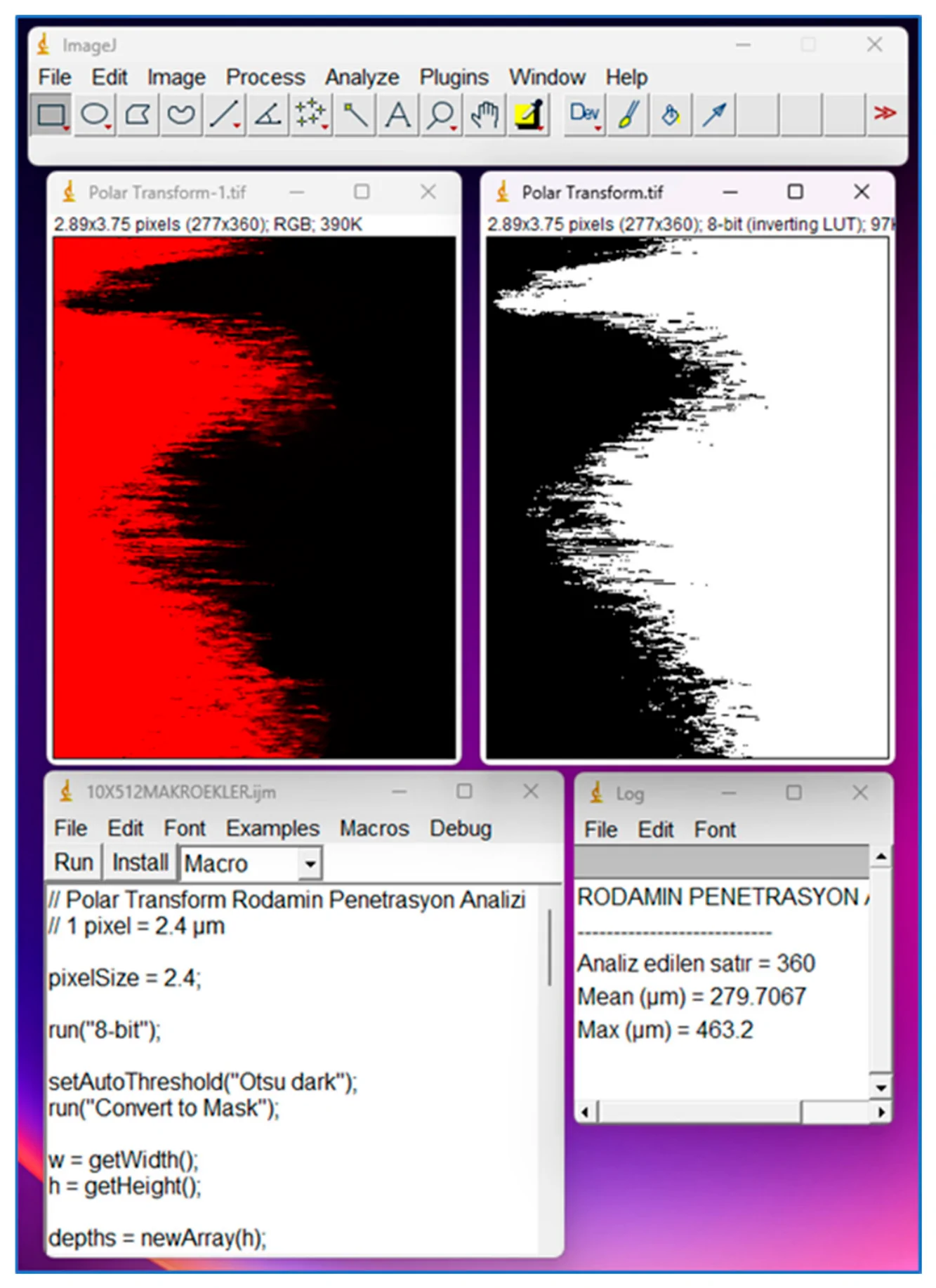
Representative screenshot of dentinal tubule penetration measurement using the custom ImageJ macro (.ijm).

A polar transform was applied to the circular CLSM images using the Polar Transformer plugin in ImageJ (Figure 4), with each row corresponding to a 1° angular position. The polar-transformed images were automatically analyzed using a custom macro prepared in ImageJ Macro (.ijm) format (Supplementary File S1). During the analysis, the images were converted to 8-bit format, and the Otsu automatic thresholding method was applied uniformly to all images to identify the fluorescent signal. Following thresholding, the images were converted into binary masks. For each row corresponding to a 1° angular position, the macro automatically identified the farthest pixel reached by the fluorescent signal from the canal wall and converted the penetration distance into micrometers using a calibration value of 2.4 µm/pixel. Angular positions with no detectable fluorescent penetration were assigned a value of 0 µm. The mean of the penetration values obtained from all evaluated angular positions was calculated as the mean penetration distance, whereas the highest value obtained among all angular positions was calculated as the maximum penetration distance. Therefore, mean penetration depth represents the average penetration across the evaluated canal circumference, including both areas with and without detectable fluorescent penetration.

The same image processing, thresholding, and measurement algorithm was uniformly applied to all specimens. The processed images and measurement outputs generated by the macro were visually inspected to verify appropriate identification of the fluorescent penetration areas and the absence of apparent segmentation or measurement errors. Because thresholding and penetration measurements were performed automatically using the same predefined macro and processing parameters for all specimens, operator-dependent variability during quantitative measurement was minimized.

### 2.6. Statistical Analysis

The data were analyzed using IBM SPSS version 23 (IBM Corp, Armonk, NY, USA). Generalized Estimating Equations (GEEs) were used to analyze the mean and maximum dentinal tubule penetration values to account for the dependence among repeated measurements obtained from the apical, middle, and coronal regions of the same tooth. A normal distribution with an identity link function was specified, and an exchangeable working correlation structure was used to account for within-tooth correlations. Tooth ID was defined as the subject variable, and root canal region (apical, middle, and coronal) as the within-subject repeated factor. Robust covariance estimates were used to estimate standard errors and covariance parameters. Canal curvature, coronal flaring, and root canal region were included in the model as main factors, and their two-way and three-way interactions were evaluated. Bonferroni correction was applied for multiple comparisons. Descriptive statistics for quantitative variables were presented as mean ± standard deviation. The level of statistical significance was set at *p* < 0.05.

In addition, baseline canal curvature angles were compared between the coronal flaring and non-flaring subgroups within each curvature category to assess the comparability of the experimental groups. Normality was assessed using the Shapiro–Wilk test. An independent-samples *t*-test was used for the straight canal subgroups, whereas the Mann–Whitney U test was used for the curved canal subgroups because the normality assumption was not satisfied.

## 3. Results

The initial canal curvature angles were comparable between the coronal flaring and non-flaring subgroups within both curvature categories. In straight canals, the mean curvature angles were 8.25 ± 1.10° and 8.25 ± 1.06° in the flaring and non-flaring subgroups, respectively (*p* = 1.000). In curved canals, the median curvature angles were 27.8° (IQR: 26.0–33.4) and 28.4° (IQR: 26.4–34.2), respectively (*p* = 0.651). The corresponding descriptive data and statistical comparisons are presented in Supplementary Table S1.

In this in vitro study, the mean and maximum penetration distances of the Rhodamine B-labeled irrigation solution into the dentinal tubules were measured, and the obtained data are presented in Table 1, Table 2, Table 3 and Table 4.

According to the GEE analysis, only the region factor had a statistically significant effect on mean penetration values (*p* < 0.001). In contrast, canal curvature (*p* = 0.073), coronal flaring (*p* = 0.151), canal curvature × coronal flaring (*p* = 0.652), canal curvature × region (*p* = 0.413), coronal flaring × region (*p* = 0.507), and canal curvature × coronal flaring × region (*p* = 0.887) interactions had no statistically significant effect on mean penetration values (Table 1).

GEE analysis showed that the root canal region had a significant effect on mean dentinal tubule penetration (*p* < 0.001). In contrast, the main effects of canal curvature (*p* = 0.073) and coronal flaring (*p* = 0.151), as well as all two-way and three-way interactions (*p* > 0.05), were not statistically significant (Table 2). Mean penetration values were 32.60 µm in the apical, 97.24 µm in the middle, and 196.46 µm in the coronal region, with significant differences among all three regions (*p* < 0.001) (Table 2). Detailed estimated marginal means, 95% Wald confidence intervals, and pairwise comparisons for mean dentinal tubule penetration are provided in Supplementary Tables S2 and S3.

According to the GEE analysis, canal curvature (*p* = 0.010), coronal flaring (*p* = 0.002), and region (*p* < 0.001) had significant effects on maximum penetration values. In contrast, canal curvature × coronal flaring (*p* = 0.851), canal curvature × region (*p* = 0.698), coronal flaring × region (*p* = 0.105), and canal curvature × coronal flaring × region (*p* = 0.817) interactions had no statistically significant effect on maximum penetration values (Table 3).

For maximum dentinal tubule penetration, the estimated marginal mean difference between straight and curved canals was 85.23 µm (95% Wald CI: 20.22–150.24; *p* = 0.010), indicating greater maximum penetration in straight canals. Similarly, the estimated marginal mean difference between specimens with and without coronal flaring was 104.10 µm (95% Wald CI: 39.09–169.12; *p* = 0.002), indicating greater maximum penetration following coronal flaring. Detailed estimated marginal means, 95% Wald confidence intervals, and pairwise comparisons for the significant main effects on maximum dentinal tubule penetration are provided in Supplementary Tables S4–S9.

Examination of the descriptive statistics showed that the maximum penetration value was 393.39 µm in curved canals, 478.62 µm in straight canals, 488.06 µm in specimens with coronal flaring, and 383.96 µm in specimens without coronal flaring. According to the regions, the maximum penetration value was 167.37 µm in the apical region, 390.82 µm in the middle region, and 749.83 µm in the coronal region.

According to the multiple comparison results, the apical, middle, and coronal regions were all statistically significantly different from each other, and the maximum penetration value was observed to increase progressively from the apical region toward the middle and coronal regions (Table 4).

## 4. Discussion

According to the findings of the present study, the root canal region was the only factor that significantly affected mean dentinal tubule penetration, whereas maximum penetration was significantly affected by root canal curvature, coronal flaring, and root canal region. Coronal flaring did not significantly alter mean dentinal tubule penetration but increased the maximum penetration depth. Maximum penetration was lower in curved canals and in the apical region.

The anatomical structure of the root canal significantly affects the success of endodontic treatment. Root canal curvature, which is one of the anatomical challenges, has been reported to make it difficult to maintain the working length during preparation by limiting access to the apical region, and also to increase the risk of procedural errors such as canal transportation, ledge formation, perforation, and instrument separation [41]. These complications may subsequently interfere with effective irrigation, disinfection, and obturation of the root canal system, potentially allowing microorganisms to persist in inadequately treated areas. Since persistent intraradicular infection is a major cause of unfavorable endodontic outcomes, the technical limitations associated with severe root canal curvature may indirectly contribute to treatment failure. Therefore, careful assessment of root canal curvature and the use of appropriate shaping and irrigation strategies are clinically important for minimizing procedural errors and optimizing disinfection, particularly in the apical region [42,43].

It has been reported that coronal flaring before preparation in curved root canals contributes to more accurate determination of the working length by allowing instruments to advance more easily within the canal, reduces apical transportation, and helps prevent iatrogenic errors such as ledge and zip formation [44,45]. Approaches such as coronal flaring, crown-down preparation, and glide path creation are recommended for safe and successful preparation in curved root canals. These procedures reduce file stress, help preserve canal anatomy, and reduce the risk of iatrogenic complications [46].

In addition, root canal curvature is one of the important anatomical factors affecting the flow characteristics of the irrigation solution within the canal and the cleaning forces generated on the canal walls. In a study in which computational fluid dynamics analysis based on real root canal anatomy was performed, it was reported that root canal curvature significantly affected the flow characteristics of the irrigation solution and made it difficult to achieve effective irrigation [47].

In a study evaluating different apical preparation diameters and tapers in curved root canals, it was reported that increasing the preparation diameter and taper improved irrigation efficacy in the apical third and provided successful results in smear layer removal [48].

In contrast, a randomized controlled clinical study conducted in teeth with necrotic pulps and apical periodontitis reported that there was no significant difference between 25/.04 and 35/.04 apical preparations in terms of reducing the bacterial load [49]. This finding also indicates that larger apical preparations may not always provide an additional disinfection advantage when activated irrigation systems are used. In another in vitro study, it was demonstrated that coronal flaring increased the penetration of the irrigation solution throughout the root canal and that this effect was particularly more pronounced in canals with smaller apical preparation diameters (#20, #25, and #30) [24]. In accordance with these findings, the apical preparation size was standardized to 25/.04 in all specimens in this study, and the effect of coronal flaring on the penetration of irrigation solutions into dentinal tubules was investigated. This approach allows the effect of coronal flaring to be evaluated independently of the apical preparation diameter with respect to the numerical differences observed in this study.

Coronal preparation provides important advantages in terms of irrigation efficacy, determination of working length, and canal shaping; however, when performed excessively, it may cause a reduction in cervical dentin thickness. During preparation, particularly when conventional instruments such as Gates-Glidden burs are used, it may lead to a reduction in the fracture resistance of the tooth [50,51]. In a study in which coronal flaring was performed using different systems, Gates-Glidden burs caused the highest crack formation in dentin, whereas the use of ProTaper Universal SX, OneFlare, and HyFlex EDM showed crack rates that were statistically similar to the control group and significantly lower [51]. Therefore, it is recommended that coronal flaring be performed using a conservative approach that preserves root dentin, taking into consideration the tooth anatomy and the instrument to be used. In this study, the coronal flaring procedure was performed using the OneFlare rotary file system.

In the literature, PIPS and SWEEPS have been reported to provide more successful results than other irrigation activation methods in smear layer removal from root canals prepared conservatively, and the effectiveness of laser-activated irrigation, particularly in the apical region, has been demonstrated [18]. These findings support that laser activation may increase irrigation efficacy in minimal preparation protocols. In a study using micro-CT, SWEEPS was shown to be statistically significantly more effective than both PIPS and ultrasonically activated irrigation in removing debris accumulated in the root canal system of mandibular molars [52]. In contrast, in a study evaluating the effectiveness of irrigation activation methods in the presence of a fractured file within the canal, it was reported that SWEEPS showed no statistically significant difference compared with PUI in the removal of debris and smear layer apical to the fractured file [53].

Current reviews indicate that laser-activated irrigation systems, including SWEEPS, have the potential to enhance the penetration of the irrigation solution into complex canal anatomy and increase irrigant exchange. However, it is emphasized that irrigation efficacy depends not only on the activation method used but also on fluid dynamics, canal anatomy, and the overall irrigation protocol applied [54]. In this study, considering that root canal curvature, which represents one of the anatomical challenges, may affect irrigation dynamics, it was considered that coronal flaring performed together with minimal apical preparation at different degrees of curvature and the SWEEPS irrigation activation method could have an effect on penetration into the dentinal tubules. Coronal flaring was intended to expand the reservoir space within the canal system, thereby allowing the irrigation solution to move more effectively through the powerful photoacoustic flow and shock waves generated by SWEEPS.

In a study evaluating the effect of pre-endodontic build-up (PEB) on the penetration of irrigation solutions into dentinal tubules using CLSM, irrigant penetration was reported to be statistically significantly increased at all canal levels in the groups with PEB compared with the groups without PEB. In addition, in the presence of PEB, higher penetration values were obtained with sonic irrigation activation, particularly in the apical third, and the most pronounced increase was observed in the PEB group using composite resin [35]. It is considered that the creation of a more controlled irrigant reservoir in the coronal region with PEB may contribute to more effective movement of the irrigation solution within the canal and increased penetration into the dentinal tubules. These findings indicate that changes in the coronal region may affect the fluid dynamics occurring during irrigation.

In another in vitro study investigating the effects of access cavity configuration and irrigation activation methods on smear layer removal, it was demonstrated that irrigation efficacy depends not only on the activation method but also on the cavity design, which facilitates the delivery of the irrigation solution into the canal system. In that study, smear layer removal in the apical third was statistically significantly higher in the group in which a traditional access cavity with a wider access cavity design was combined with SWEEPS activation compared with the other groups [55]. These findings suggest that coronal flaring may increase the movement of the irrigation solution within the root canal system. Similarly, in this study, the effect of the interaction between coronal flaring and root canal region on dentinal tubule penetration was evaluated. Although numerical increases in dentinal tubule penetration values were observed, particularly in the apical region, in groups with straight and curved root canals subjected to coronal flaring, the coronal flaring × region interaction was not statistically significant.

According to the findings obtained in this study, only the region factor had a statistically significant effect on the mean penetration distance of the irrigation solution into the dentinal tubules in straight and curved root canals. The mean penetration distances measured in the coronal, middle, and apical thirds differed significantly from each other; the highest penetration was observed in the coronal region, followed by the middle and apical regions, respectively. Consistent with this study, a study evaluating the penetration of Rhodamine B-labeled NaOCl into dentinal tubules using CLSM reported that, with all activation methods, the mean penetration distance of the irrigation solution was highest in the coronal third, lower in the middle third, and lowest in the apical third [56]. Similarly, a study evaluating the penetration of CHX into dentinal tubules using CLSM reported that the penetration depth was highest in the coronal third, lower in the middle third, and lowest in the apical third [57]. This regional difference is thought to be associated with the decrease in the diameter and density of dentinal tubules from the coronal to the apical region, as well as the greater difficulty of the irrigation solution in reaching this region.

In the present study, although a numerical increase was observed in the mean penetration values of the irrigation solution into the dentinal tubules in groups with straight and curved root canals subjected to coronal flaring, this increase was not statistically significant. In contrast, canal curvature and coronal flaring alone were found to have statistically significant effects on maximum penetration depth in this study. Maximum penetration depth was significantly higher in straight root canals than in curved canals and in specimens subjected to coronal flaring than in those without coronal flaring. Although there are studies in the literature evaluating the maximum penetration depth of irrigation solutions into dentinal tubules [30,49], no studies have been found investigating the effects of canal curvature, coronal flaring, and root canal region on irrigation efficacy in terms of two-way and three-way interactions. Therefore, direct comparison of the interaction findings regarding maximum penetration depth obtained in this study with the literature remains limited.

Mean and maximum penetration depths represent different but complementary aspects of irrigant penetration. In the present study, mean penetration depth was calculated across all evaluated angular positions, including a value of 0 µm for positions without detectable fluorescence, and therefore reflects penetration across the evaluated canal circumference. In contrast, maximum penetration depth represents the deepest penetration observed at a single angular position and reflects the greatest localized depth reached by the fluorescently labeled irrigant. This distinction may explain why canal curvature and coronal flaring significantly affected maximum penetration but not mean penetration. These factors may therefore influence the deepest localized penetration without producing a consistent change in penetration across the evaluated canal circumference. However, because maximum penetration is based on the single deepest measurement, it may be more sensitive to local dentinal anatomy, localized fluorescence, and isolated high values and should therefore be interpreted cautiously. The hypothesis of this study was partially rejected.

The present study has some limitations. Limitations include the inability of in vitro conditions to fully reflect the clinical environment and the use of only a single tooth type, specifically the distobuccal canals of the upper first molars. Although all specimens were obtained from adult patients aged 18–60 years, the exact age associated with each individual tooth was not available. Therefore, it should be considered that differences may exist among the specimens in terms of dentinal tubule diameter, density, and degree of calcification.

Another limitation of the present study is that the actual geometric changes produced by coronal flaring were not quantitatively assessed. Although the intended coronal enlargement was standardized according to the apical diameter and taper of the OneFlare instrument and its predefined insertion depth, the actual amount and volume of dentin removed were not measured using micro-computed tomography or another three-dimensional imaging method. Similarly, although one of the proposed effects of coronal flaring is the modification of root canal geometry, including a potential reduction in canal curvature, postoperative curvature was not reassessed. The present study was designed to evaluate the effect of applying a standardized coronal flaring procedure on irrigant penetration rather than to quantify the geometric changes produced by the instrument. Therefore, although changes in canal geometry resulting from coronal flaring may have contributed to the observed penetration outcomes, the magnitude of these dimensional changes, including curvature reduction, and their specific relationship with dentinal tubule penetration could not be determined.

Another limitation of this study is that the same irrigation solution and a standardized SWEEPS activation protocol were used in all specimens. Accordingly, the effects of coronal flaring and canal curvature on the penetration of the irrigation solution into the dentinal tubules were evaluated only under these experimental conditions, and no inference can be made regarding the results that may be obtained when different irrigation solutions or activation methods are used. Although Rhodamine B-labeled NaOCl has been used in previous CLSM studies to evaluate irrigant penetration, CLSM detects Rhodamine B fluorescence rather than NaOCl itself. Furthermore, the chemical and fluorescence stability of Rhodamine B in 5.25% NaOCl was not independently assessed under the specific experimental conditions of the present study. Therefore, the penetration measurements should be interpreted as the distribution of the Rhodamine B-labeled NaOCl solution rather than as direct detection of NaOCl [36].

The Rhodamine B-labeled NaOCl solution was applied after completion of the standardized activated final irrigation protocol. This sequence, in accordance with the literature, was selected to evaluate fluorescent tracer penetration after final canal cleaning and was applied identically to all experimental groups. Nevertheless, the preceding SWEEPS-activated EDTA and NaOCl irrigation may have modified dentinal tubule accessibility and thereby influenced the subsequent penetration values. Therefore, although this factor did not differ among the experimental groups, its contribution to the absolute penetration values could not be independently determined [35,36].

Accordingly, further in vitro and clinical studies involving different sample sizes, different canal morphologies, and various irrigation activation protocols are considered necessary to more comprehensively demonstrate the effect of coronal flaring on the effectiveness of laser-activated irrigation.

## 5. Conclusions

Under the present experimental conditions, coronal flaring was not significantly associated with mean dentinal tubule penetration but was significantly associated with greater maximum localized penetration depth. Maximum penetration was also higher in straight root canals than in curved canals. Both mean and maximum penetration showed the highest values in the coronal region and the lowest values in the apical region.

From a clinical perspective, these findings suggest that root canal curvature and coronal flaring may influence the ability of SWEEPS laser-activated irrigation solutions to penetrate dentinal tubules, particularly in terms of maximum penetration. The lower maximum penetration observed in curved canals and in the apical region highlights the potential challenges associated with delivering irrigation solutions to anatomically difficult areas. Therefore, shaping and irrigation activation procedures, as well as root canal anatomy, should be considered to optimize irrigation solution penetration while maintaining a conservative approach to dentin removal. Further studies involving different canal anatomies and irrigation activation protocols to support the clinical applicability of these findings will contribute to a more comprehensive evaluation of the effect of coronal flaring on irrigant penetration.

## Figures and Tables

**Table 1:** Comparison of the effects of canal curvature, coronal flaring, and region factors on mean penetration values.

	Test Statistics	*p* ^x^
Canal Curvature	3.224	0.073
Coronal Flaring	2.058	0.151
Region	450.252	**<0.001**
Canal Curvature × Coronal Flaring	0.203	0.652
Canal Curvature × Region	1.766	0.413
Coronal Flaring × Region	1.360	0.507
Canal Curvature × Coronal Flaring × Region	0.239	0.887

^x^ Generalized Estimating Equations (GEEs). Bold values indicate statistical significance (*p* < 0.05).

**Table 2:** Descriptive statistics and multiple-comparison results for mean penetration values according to the effects of canal curvature, coronal flaring, and region factors (µm).

Region	Coronal Flaring	Canal Curvature		Total
		Curved Canals	Straight Canals	
Apical	With	34.31 ± 13.16	39.7 ± 10.48	37 ± 12.05
	Without	20.24 ± 2.08	36.14 ± 16.08	28.19 ± 13.88
	Total	27.27 ± 11.71	37.92 ± 13.51	32.6 ± 13.65 ^a^
Middle	With	98.47 ± 19	116.34 ± 88.89	107.4 ± 64.05
	Without	71.56 ± 38.49	102.6 ± 53.78	87.08 ± 48.74
	Total	85.01 ± 32.9	109.47 ± 72.8	97.24 ± 57.45 ^b^
Coronal	With	194.7 ± 76.78	206.88 ± 79.35	200.79 ± 77.26
	Without	185.99 ± 28.29	198.25 ± 85.62	192.12 ± 63.2
	Total	190.35 ± 57.24	202.57 ± 81.54	196.46 ± 70.24 ^c^
Total	With	109.16 ± 80.55	120.97 ± 96.7	115.07 ± 88.8
	Without	92.59 ± 74.97	112.33 ± 88.75	102.46 ± 82.38
	Total	100.88 ± 77.91	116.65 ± 92.5	

Mean ± standard deviation; a–c: there is no difference between regions with the same letter.

**Table 3:** Comparison of the effects of canal curvature, coronal flaring, and region factors on maximum penetration values.

	Test Statistics	*p* ^x^
Canal Curvature	6.602	**0.010**
Coronal Flaring	9.85	**0.002**
Region	254.126	**<0.001**
Canal Curvature × Coronal Flaring	0.035	0.851
Canal Curvature × Region	0.718	0.698
Coronal Flaring × Region	4.505	0.105
Canal Curvature × Coronal Flaring × Region	0.403	0.817

^x^ Generalized Estimating Equations (GEEs). Bold values indicate statistical significance (*p* < 0.05).

**Table 4:** Descriptive statistics and multiple comparison results for maximum penetration values according to the effects of canal curvature, coronal flaring, and region factors (µm).

Region	Coronal Flaring	Canal Curvature		Total
		Curved Canals	Straight Canals	
Apical	with	138.8 ± 79.76	239.54 ± 178.27	189.17 ± 145.47
	without	120.25 ± 25.54	170.88 ± 77.28	145.57 ± 62.3
	Total	129.53 ± 59.16	205.21 ± 139.91	167.37 ± 113.29 ^a^
Middle	with	396.29 ± 102.49	447.99 ± 334.5	422.14 ± 245.42
	without	328.59 ± 134.31	390.41 ± 223.89	359.5 ± 184.78
	Total	362.44 ± 122.73	419.2 ± 282.26	390.82 ± 218.06 ^b^
Coronal	with	791.91 ± 276.53	913.83 ± 461.84	852.87 ± 380.5
	without	584.51 ± 262.21	709.08 ± 247.42	646.8 ± 259.26
	Total	688.21 ± 285.82	811.46 ± 379.88	749.83 ± 339.62 ^c^
Total	with	442.33 ± 321.62	533.79 ± 442.29	488.06 ± 387.7
	without	344.45 ± 254.56	423.46 ± 295.64	383.96 ± 277.49
	Total	393.39 ± 292.9	478.62 ± 378.59	

Mean ± standard deviation; a–c: there is no difference between regions with the same letter.

## Data Availability

The data supporting the findings of this study are provided within the article. Further information may be obtained from the corresponding author upon reasonable request.

## Supplementary Materials

The following supporting information can be downloaded at: https://www.mdpi.com/article/10.3390/medicina62091737/s1, Table S1: Comparison of initial canal curvature angles between the coronal flaring and non-flaring subgroups; Table S2: Average differences in penetrations and multiple comparison results; Table S3: Estimated average values and confidence intervals for penetration; Table S4: Estimated average values and confidence intervals for the canal curvature; Table S5: Average differences and comparison results related to canal curvature; Table S6: Estimated mean values and confidence intervals for the flaring factor; Table S7: Average differences and multiple comparison results related to flaring; Table S8: Estimated mean values and confidence intervals for the Penetration (Region) factor; Table S9: Average differences and multiple comparison results of penetration factor; File S1: ImageJ macro (.ijm) used for the automated measurement of dentinal tubule penetration.

## Abbreviations

The following abbreviations are used in this manuscript:

SWEEPS	Shock Wave-Enhanced Emission Photoacoustic Streaming
PIPS	Photon-Induced Photoacoustic Streaming
CLSM	Confocal Laser Scanning Microscopy
NaOCl	Sodium Hypochlorite
EDTA	Ethylenediaminetetraacetic Acid
GEEs	Generalized Estimating Equations
PEB	Pre-Endodontic Build-Up
