# Supplementary material for: Evaluation of the Effect of Coronal Flaring on SWEEPS Laser-Activated Irrigation in Root Canals with Different Curvatures: A Confocal Laser Scanning Microscopy Study
Source: Medicina (Kaunas). 2026 Sep 9;62(9):1737. doi: 10.3390/medicina62091737 (PMC13609472; doi:10.3390/medicina62091737)
Supplement: Supplementary file 1 [file medicina-62-01737-s001.zip › medicina-4528330-Supplementary File S1.pdf]

ImageJ Macro We Use to Measure Average and Maximum Penetration Values on a Polar Transformed Confocal Image

```
// Polar Transform Rodamin Penetrasyon Analizi
// 1 pixel = 2.4 µm
pixelSize = 2.4;
run("8-bit");
setAutoThreshold("Otsu dark");
run("Convert to Mask");
w = getWidth();
h = getHeight();
depths = newArray(h);
n = 0;
// Find the rightmost white pixel for each row.
for (y = 0; y < h; y++) {
   depth = -1;
   for (x = w - 1; x >= 0; x--) {
      if (getPixel(x, y) > 0) {
         depth = x;
         break;
      }
   }
   if (depth >= 0) {
      depths[n] = depth * pixelSize;
      n++;
   }
}
// Transfer only the used values to the new array.
values = newArray(n);
for (i = 0; i < n; i++)
   values[i] = depths[i];
// İstatistikler
Array.getStatistics(values, min, max, mean);
print("\\Clear");
print("RHODAMINE PENETRATION ANALYSIS ");
print("------------------");
print("Analiz edilen satır = " + n);
print("Mean (µm) = " + mean);
print("Max (µm) = " + max);
if (isOpen("Results"))
   run("Clear Results");
setResult("Rows", 0, n);
setResult("Mean_um", 0, mean);
setResult("Max_um", 0, max);
updateResults();
```
