# Supplementary material for: Evaluation of the Effect of Coronal Flaring on SWEEPS Laser-Activated Irrigation in Root Canals with Different Curvatures: A Confocal Laser Scanning Microscopy Study
Source: Medicina (Kaunas). 2026 Sep 9;62(9):1737. doi: 10.3390/medicina62091737 (PMC13609472; doi:10.3390/medicina62091737)
Supplement: Supplementary file 1 [file medicina-62-01737-s001.zip › medicina-4528330-Supplementary Tables.pdf]

# Supplementary Tables

**Supplementary Table S1.** Comparison of initial canal curvature angles between the coronal flaring and non-flaring subgroups.

| Canal curvature category | Coronal flaring | n | Initial curvature angle (°) | p-value |
|---|---|---|---|---|
| **Straight** | **With flaring** | **19** | **8.25 ± 1.10** | |
| **Straight** | **Without flaring** | **19** | **8.25 ± 1.06** | **1.000[a]** |
| **Curved** | **With flaring** | **19** | **27.8 (26.0–33.4)** | |
| **Curved** | **Without flaring** | **19** | **28.4 (26.4–34.2)** | **0.651[b]** |

* Data are presented as mean ± standard deviation (SD) for normally distributed data and median (interquartile range [IQR]) for non-normally distributed data. [a] Independent-samples t-test; [b] Mann–Whitney U test. Statistical significance was set at $p < 0.05$.

**Supplementary Table S2.** Average differences in penetrations and multiple comparison results

| (I) Penetration | (J) Penetration | Mean Difference (I-J) | Std. Error | df | Bonferroni Sig. | 95% Wald Confidence Interval for Difference | |
|---|---|---|---|---|---|---|---|
| | | | | | | Lower | Upper |
| Apical | Middle | -64.6453[a] | 5.93046 | 1 | .000 | -78.8427 | -50.4479 |
| | Coronal | -163.8606[a] | 7.72864 | 1 | .000 | -182.3628 | -145.3584 |
| Middle | Apical | 64.6453[a] | 5.93046 | 1 | .000 | 50.4479 | 78.8427 |
| | Coronal | -99.2153[a] | 6.68468 | 1 | .000 | -115.2183 | -83.2123 |
| Coronal | Apical | 163.8606[a] | 7.72864 | 1 | .000 | 145.3584 | 182.3628 |
| | Middle | 99.2153[a] | 6.68468 | 1 | .000 | 83.2123 | 115.2183 |

Pairwise comparisons of estimated marginal means based on the original scale of dependent variable Mean

a. The mean difference is significant at the .05 level.

**Supplementary Table S3.** Estimated average values and confidence intervals for penetration.

**Estimates**

| Penetration | Mean | Std. Error | 95% Wald Confidence Interval | |
|---|---|---|---|---|
| | | | Lower | Upper |
| Apical | 32.5950 | 130427 | 30.0387 | 35.1513 |
| Middle | 97.2403 | 6.27564 | 84.9403 | 109.5403 |
| Koronal | 196.4556 | 7.95811 | 180.8580 | 212.0532 |

**Supplementary Table S4.** Estimated average values and confidence intervals for the canal curvature

**Estimates**

| Canal Curvature | Mean | Std. Error | 95% Wald Confidence Interval | |
|---|---|---|---|---|
| | | | Lower | Upper |
| Curved | 393.3912 | 17.42516 | 359.2385 | 427.5439 |
| Straight | 478.6224 | 28.22463 | 423.3031 | 533.9416 |

**Supplementary Table S5.** Average differences and comparison results related to canal curvature

**Pairwise Comparisons**

| (I)Canal curvature | (J)Canal curvature | Mean Difference (I-J) | Std. Error | df | Bonferroni Sig. | 95% Wald Confidence Interval for Difference | |
|---|---|---|---|---|---|---|---|
| | | | | | | Lower | Upper |
| Curved | Straight | -85.2311[a] | 33.17026 | 1 | .010 | -150.2436 | -20.2186 |
| Straight | Curved | 85.2311[a] | 33.17026 | 1 | .010 | 20.2186 | 150.2436 |

Pairwise comparisons of estimated marginal means based on the original scale of dependent variable Max

a. The mean difference is significant at the .05 level.

**Supplementary Table S6.** Estimated mean values and confidence intervals for the flaring factor.

**Estimates**

| Flaring | Mean | Std. Error | 95% Wald Confidence Interval | |
|---|---|---|---|---|
| | | | Lower | Upper |
| With | 488.0583 | 27.19168 | 434.7636 | 541.3531 |
| Without | 383.9553 | 18.99680 | 346.7222 | 421.1883 |

**Supplementary Table S7.** Average differences and multiple comparison results related to flaring.

**Pairwise Comparisons**

| (I) Flaring | (J) Flaring | Mean Difference (I-J) | Std. Error | df | Bonferroni Sig. | 95% Wald Confidence Interval for Difference | |
|---|---|---|---|---|---|---|---|
| | | | | | | Lower | Upper |
| With | Without | 104.1031[a] | 33.17026 | 1 | .002 | 39.0906 | 169.1156 |
| Without | with | -104.1031[a] | 33.17026 | 1 | .002 | -169.1156 | -39.0906 |

Pairwise comparisons of estimated marginal means based on the original scale of dependent variable Max

a. The mean difference is significant at the .05 level.

**Supplementary Table S8.** Estimated mean values and confidence intervals for the Penetration (Region) factor.

Estimated mean values and confidence intervals for the Penetration (Region) factor.

**Estimates**

| Penetration | Mean | Std. Error | 95% Wald Confidence Interval | |
|---|---|---|---|---|
| | | | Lower | Upper |
| Apical | 167.3684 | 11.81119 | 144.2189 | 190.5179 |
| Middle | 390.8197 | 24.36937 | 343.0566 | 438.5828 |
| Coronal | 749.8322 | 36.16687 | 678.9465 | 820.7180 |

**Supplementary Table S9.** Average differences and multiple comparison results of penetration factor

**Pairwise Comparisons**

| (I) Penetration | (J) Penetration | Mean Difference (I-J) | Std. Error | df | Bonferroni Sig. | 95% Wald Confidence Interval for Difference | |
|---|---|---|---|---|---|---|---|
| | | | | | | Lower | Upper |
| Apical | Middle | -223.4513[a] | 24.71893 | 1 | .000 | -282.6279 | -164.2747 |
| | Coronal | -582.4638[a] | 38.47287 | 1 | .000 | -674.5671 | -490.3605 |
| Middle | Apical | 223.4513[a] | 24.71893 | 1 | .000 | 164.2747 | 282.6279 |
| | Koronal | -359.0125[a] | 39.46459 | 1 | .000 | -453.4899 | -264.5351 |
| Coronal | Apical | 582.4638[a] | 38.47287 | 1 | .000 | 490.3605 | 674.5671 |
| | Middle | 359.0125[a] | 39.46459 | 1 | .000 | 264.5351 | 453.4899 |

Pairwise comparisons of estimated marginal means based on the original scale of dependent variable Max

a. The mean difference is significant at the .05 level.
